# 2,4,6-Tri­nitro­phenyl furan-2-carboxyl­ate

**DOI:** 10.1107/S1600536813028274

**Published:** 2013-10-23

**Authors:** Rodolfo Moreno-Fuquen, Fabricio Mosquera, Alan R. Kennedy

**Affiliations:** a Departamento de Química – Facultad de Ciencias, Universidad del Valle, Apartado 25360, Santiago de Cali, Colombia; b WestCHEM, Department of Pure and Applied Chemistry, University of Strathclyde, 295 Cathedral Street, Glasgow G1 1XL, Scotland

## Abstract

In the title carboxyl­ate derivative, C_11_H_5_N_3_O_9_, the picryl ring forms an angle of 75.79 (7)° with the ester fragment, indicating a near perpendicular disposition. The nitro substituents are variously oriented with respect to the picryl ring [dihedral angles = 3.22 (10), 16.03 (12) and 36.63 (10)°]. In the crystal, mol­ecules form helical chains sustained by C—H⋯O inter­actions along [010]. The furanyl residue is disordered, having two coplanar slightly displaced orientations [major component = 0.730 (9)].

## Related literature

For similar esters, see: Moreno-Fuquen *et al.* (2012 ▶, 2013 ▶). For hydrogen bonding, see: Nardelli (1995 ▶).
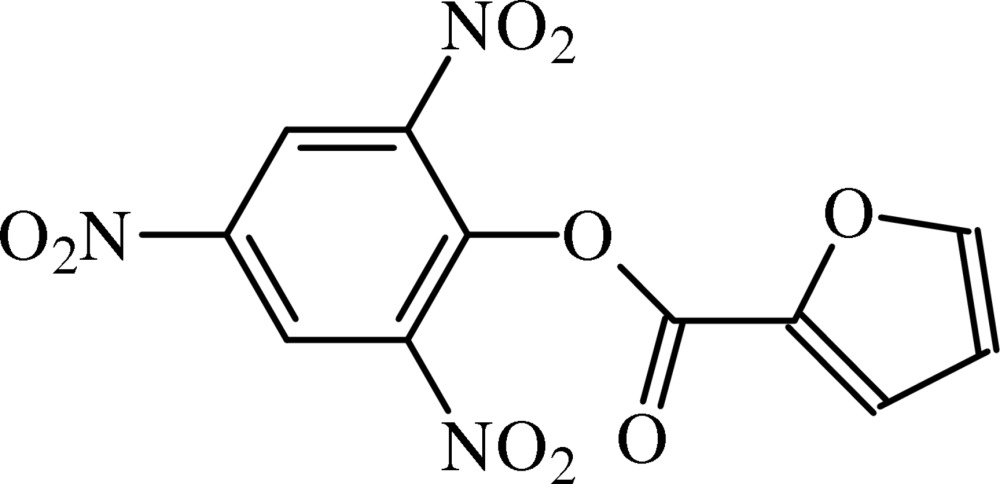


## Experimental

### 

#### Crystal data

C_11_H_5_N_3_O_9_
*M* *_r_* = 323.18
Orthorhombic, 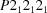

*a* = 7.0982 (3) Å
*b* = 8.4931 (4) Å
*c* = 20.4970 (9) Å
*V* = 1235.68 (10) Å^3^
*Z* = 4
Mo *K*α radiation
μ = 0.16 mm^−1^
*T* = 123 K
0.35 × 0.22 × 0.11 mm

#### Data collection

Oxford Diffraction Xcalibur E diffractometer
4861 measured reflections
2669 independent reflections
2395 reflections with *I* > 2σ(*I*)
*R*_int_ = 0.022

#### Refinement

*R*[*F* ^2^ > 2σ(*F* ^2^)] = 0.039
*wR*(*F* ^2^) = 0.095
*S* = 1.06
2669 reflections
224 parameters
12 restraints
H-atom parameters constrained
Δρ_max_ = 0.27 e Å^−3^
Δρ_min_ = −0.27 e Å^−3^

### 

Data collection: *CrysAlis PRO* (Oxford Diffraction, 2010 ▶); cell refinement: *CrysAlis PRO*; data reduction: *CrysAlis PRO*; program(s) used to solve structure: *SHELXS97* (Sheldrick, 2008 ▶); program(s) used to refine structure: *SHELXL97* (Sheldrick, 2008 ▶); molecular graphics: *ORTEP-3 for Windows* (Farrugia, 2012 ▶) and *Mercury* (Macrae *et al.*, 2006 ▶); software used to prepare material for publication: *WinGX* (Farrugia, 2012 ▶).

## Supplementary Material

Crystal structure: contains datablock(s) I, global. DOI: 10.1107/S1600536813028274/tk5263sup1.cif

Structure factors: contains datablock(s) I. DOI: 10.1107/S1600536813028274/tk5263Isup2.hkl

Supplementary material file. DOI: 10.1107/S1600536813028274/tk5263Isup3.cml

Additional supplementary materials:  crystallographic information; 3D view; checkCIF report

## Figures and Tables

**Table 1:** Hydrogen-bond geometry (Å, °)

*D*—H⋯*A*	*D*—H	H⋯*A*	*D*⋯*A*	*D*—H⋯*A*
C5—H5⋯O8^i^	0.95	2.32	3.270 (2)	180

Symmetry code: (i) 
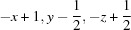
.

**Fig. 1.**
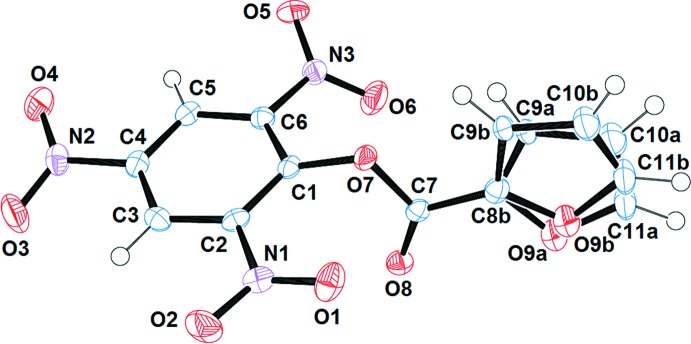
Molecular conformation and atom numbering scheme for the title compound with displacement ellipsoids drawn at the 50% probability level. H atoms are shown as spheres of arbitrary radius. In the disordered furanyl residue, the atoms labelled with an "a" have site occupancy factors of 0.730 (9).

**Fig. 2.**
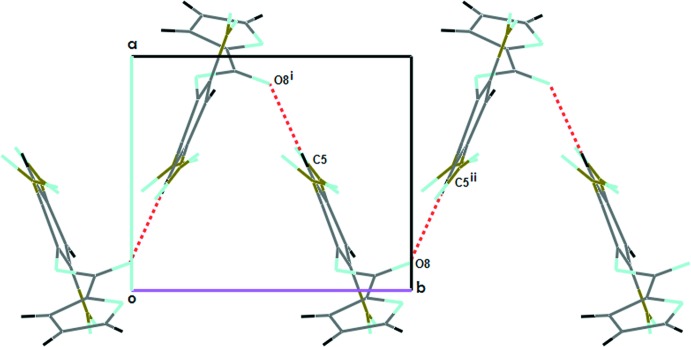
Part of the crystal structure of (I), showing the formation of helical chains which running along [010]. Symmetry code: (i) -x, +y-1/2, -z+1/2. The C—H···O interactions are shown as dashed lines.

## supplementary crystallographic information

### 1. Comment

In the present work, the structure of the 2,4,6-trinitrophenyl furan 2-carboxylate (I) has been determined as a part of an in-depth study of picryl substituted-esters carried out in our research group. Descriptions of similar structures have been published recently: 2,4,6-trinitrophenyl 3-chlorobenzoate (Moreno-Fuquen *et al.*, 2013), and 2,4,6-trinitrophenyl benzoate (Moreno-Fuquen *et al.*, 2012). The molecular structure of (I) is shown in Fig. 1. Bond distances and angles agree with the molecular features exhibited by other picryl substituted-esters, as described in detail in previous work (Moreno-Fuquen *et al.*, 2012 and 2013). The picryl ring forms an angle of 75.79 (7)° with the ester fragment. The nitro groups form dihedral angles with the adjacent benzene ring of 3.22 (10), 16.03 (12) and 36.63 (10)° for O1—N1—O2, O3—N2—O4 and O5—N3—O6, respectively. The atoms at the furanyl ring are disordered over two positions with occupancies refined to 0.730 (9) and 0.270 (9) for C8A–C11A/O9A and C8B–C11B/O9B, respectively. Appropriate restraints were required (see experimental section) to give chemically acceptable geometries for these fragments. In the crystal the molecules are linked by weak C—H···O interactions, forming one-dimensional helical chains running along [010], as shown in Fig. 2 & Table 1. The C5 atom of the benzene ring at (*x*, *y*, *z*) acts as a hydrogen-bond donor to carbonyl atom O8 at (-*x*+1, +*y*-1/2, -*z*+1/2) (see Nardelli, 1995).

### 2. Experimental

The reagents and solvents for the synthesis were obtained from the Aldrich Chemical Co., and were used without additional purification. The title molecule was synthesized using equimolar quantities of 2-furoyl chloride (0.252 g, 1.931 mmol) and picric acid (0.442 g). The reagents were dissolved in acetonitrile and the solution was taken to reflux for about an hour. A pale-yellow solid was obtained after leaving the solvent to evaporate. The solid was washed with distilled water and cold methanol to eliminate impurities. Crystals of good quality and suitable for single-crystal X-ray diffraction were grown from its acetonitrile solution. IR spectra were recorded on a FT—IR SHIMADZU IR-Affinity-1 spectrophotometer. Pale Yellow crystals; yield 52%; m.p 383 (1) K. IR (KBr) 3088.17 cm^-1^ (aromatic C—H); 1764.94 cm^-1^ (ester C=O); 1544.08 cm^-1^, 1343.48 cm^-1^ (–NO2); 1234.50 cm^-1^ (C(=O)—O).

### 3. Refinement

Bond lengths of the disordered furanyl ring were restrained to 1.37 (1) Å for C—O and 1.325 (20) and 1.45 (2) Å for the formally double and single C—C bonds, respectively. Restraints were also applied to force equivalence of displacement parameters for each pair of disordered atoms. All H-atoms were positioned at geometrically idealized positions with C—H distances of 0.95 Å, and with *U*_iso_(H) = 1.2*U*_eq_ of the parent C-atoms.

### Crystal data

**Table:**

C_11_H_5_N_3_O_9_	*D*_x_ = 1.737 Mg m^−^^3^
*M**_r_* = 323.18	Melting point: 435(1) K
Orthorhombic, *P*2_1_2_1_2_1_	Mo *K*α radiation, λ = 0.71073 Å
Hall symbol: P 2ac 2ab	Cell parameters from 4861 reflections
*a* = 7.0982 (3) Å	θ = 3.0–27.0°
*b* = 8.4931 (4) Å	µ = 0.16 mm^−^^1^
*c* = 20.4970 (9) Å	*T* = 123 K
*V* = 1235.68 (10) Å^3^	Block, pale-yellow
*Z* = 4	0.35 × 0.22 × 0.11 mm
*F*(000) = 656	

### Data collection

**Table:**

Oxford Diffraction Xcalibur E diffractometer	2395 reflections with *I* > 2σ(*I*)
Radiation source: fine-focus sealed tube	*R*_int_ = 0.022
Graphite monochromator	θ_max_ = 27.0°, θ_min_ = 3.0°
ω scans	*h* = −9→9
4861 measured reflections	*k* = −10→8
2669 independent reflections	*l* = −26→20

### Refinement

**Table:**

Refinement on *F*^2^	Primary atom site location: structure-invariant direct methods
Least-squares matrix: full	Secondary atom site location: difference Fourier map
*R*[*F*^2^ > 2σ(*F*^2^)] = 0.039	Hydrogen site location: inferred from neighbouring sites
*wR*(*F*^2^) = 0.095	H-atom parameters constrained
*S* = 1.06	*w* = 1/[σ^2^(*F*_o_^2^) + (0.0469*P*)^2^ + 0.1241*P*] where *P* = (*F*_o_^2^ + 2*F*_c_^2^)/3
2669 reflections	(Δ/σ)_max_ < 0.001
224 parameters	Δρ_max_ = 0.27 e Å^−^^3^
12 restraints	Δρ_min_ = −0.27 e Å^−^^3^

### Special details

**Table:**

Geometry. All esds (except the esd in the dihedral angle between two l.s. planes) are estimated using the full covariance matrix. The cell esds are taken into account individually in the estimation of esds in distances, angles and torsion angles; correlations between esds in cell parameters are only used when they are defined by crystal symmetry. An approximate (isotropic) treatment of cell esds is used for estimating esds involving l.s. planes.
Refinement. Refinement of *F*^2^ against ALL reflections. The weighted *R*-factor *wR* and goodness of fit *S* are based on *F*^2^, conventional *R*-factors *R* are based on *F*, with *F* set to zero for negative *F*^2^. The threshold expression of *F*^2^ > σ(*F*^2^) is used only for calculating *R*-factors(gt) *etc*. and is not relevant to the choice of reflections for refinement. *R*-factors based on *F*^2^ are statistically about twice as large as those based on *F*, and *R*- factors based on ALL data will be even larger.

### Fractional atomic coordinates and isotropic or equivalent isotropic displacement parameters (Å^2^)

**Table:**

	*x*	*y*	*z*	*U*_iso_*/*U*_eq_	Occ. (<1)
O1	−0.18433 (19)	0.8534 (2)	0.26898 (7)	0.0305 (4)	
O2	−0.1529 (2)	0.8749 (2)	0.37311 (8)	0.0363 (5)	
O3	0.4160 (2)	0.7394 (3)	0.48267 (7)	0.0428 (5)	
O4	0.6111 (2)	0.5845 (2)	0.43352 (8)	0.0375 (4)	
O5	0.5765 (2)	0.53627 (19)	0.19586 (8)	0.0293 (4)	
O6	0.4404 (2)	0.7359 (2)	0.15032 (7)	0.0401 (5)	
O7	0.08707 (19)	0.73046 (16)	0.19797 (6)	0.0204 (3)	
O8	0.11722 (19)	0.99352 (16)	0.18492 (7)	0.0215 (3)	
N1	−0.0930 (2)	0.8411 (2)	0.31894 (8)	0.0215 (4)	
N2	0.4759 (2)	0.6722 (2)	0.43417 (8)	0.0253 (4)	
N3	0.4676 (2)	0.6478 (2)	0.19627 (8)	0.0225 (4)	
C1	0.1840 (3)	0.7368 (2)	0.25609 (9)	0.0164 (4)	
C2	0.1026 (3)	0.7816 (2)	0.31521 (9)	0.0178 (4)	
C3	0.1979 (3)	0.7648 (3)	0.37363 (10)	0.0194 (4)	
H3	0.1425	0.7973	0.4136	0.023*	
C4	0.3760 (3)	0.6994 (2)	0.37219 (9)	0.0199 (4)	
C5	0.4643 (3)	0.6543 (2)	0.31504 (9)	0.0188 (4)	
H5	0.5859	0.6077	0.3153	0.023*	
C6	0.3678 (3)	0.6802 (2)	0.25754 (9)	0.0182 (4)	
C7	0.0627 (3)	0.8691 (2)	0.16480 (9)	0.0169 (4)	
C8A	−0.032 (7)	0.8401 (19)	0.1031 (12)	0.020 (2)	0.730 (9)
O9A	−0.0526 (9)	0.9683 (6)	0.0630 (3)	0.0319 (8)	0.730 (9)
C9A	−0.1062 (9)	0.7071 (7)	0.0776 (2)	0.0194 (10)	0.730 (9)
H9	−0.1115	0.6058	0.0972	0.023*	0.730 (9)
C10A	−0.1754 (5)	0.7519 (7)	0.0143 (2)	0.0298 (12)	0.730 (9)
H10	−0.2333	0.6848	−0.0169	0.036*	0.730 (9)
C11A	−0.1425 (7)	0.9074 (7)	0.0077 (2)	0.0316 (11)	0.730 (9)
H11	−0.1758	0.9678	−0.0296	0.038*	0.730 (9)
C8B	−0.044 (19)	0.835 (5)	0.105 (3)	0.020 (2)	0.270 (9)
O9B	−0.077 (3)	0.9350 (19)	0.0532 (8)	0.0319 (8)	0.270 (9)
C9B	−0.103 (3)	0.687 (2)	0.0960 (7)	0.0194 (10)	0.270 (9)
H9B	−0.0902	0.5978	0.1238	0.023*	0.270 (9)
C10B	−0.1880 (18)	0.6968 (18)	0.0363 (6)	0.0298 (12)	0.270 (9)
H10B	−0.2524	0.6115	0.0163	0.036*	0.270 (9)
C11B	−0.171 (2)	0.840 (2)	0.0088 (7)	0.0316 (11)	0.270 (9)
H11B	−0.2151	0.8698	−0.0332	0.038*	0.270 (9)

### Atomic displacement parameters (Å^2^)

**Table:**

	*U*^11^	*U*^22^	*U*^33^	*U*^12^	*U*^13^	*U*^23^
O1	0.0175 (7)	0.0428 (11)	0.0313 (8)	0.0018 (8)	−0.0020 (6)	0.0103 (8)
O2	0.0285 (8)	0.0485 (12)	0.0320 (9)	0.0119 (8)	0.0068 (7)	−0.0089 (9)
O3	0.0451 (10)	0.0646 (13)	0.0187 (8)	0.0105 (11)	−0.0038 (7)	−0.0073 (9)
O4	0.0357 (9)	0.0426 (11)	0.0341 (9)	0.0117 (9)	−0.0114 (8)	0.0043 (8)
O5	0.0273 (7)	0.0241 (8)	0.0364 (9)	0.0105 (7)	0.0031 (7)	−0.0032 (7)
O6	0.0455 (10)	0.0491 (11)	0.0256 (8)	0.0213 (10)	0.0083 (7)	0.0133 (9)
O7	0.0236 (7)	0.0165 (7)	0.0212 (7)	0.0004 (6)	−0.0081 (6)	−0.0001 (6)
O8	0.0218 (7)	0.0184 (8)	0.0243 (8)	−0.0004 (6)	−0.0002 (7)	−0.0004 (7)
N1	0.0178 (8)	0.0186 (8)	0.0280 (9)	−0.0005 (7)	0.0033 (7)	0.0012 (9)
N2	0.0263 (10)	0.0300 (11)	0.0196 (9)	−0.0045 (8)	−0.0051 (7)	0.0023 (9)
N3	0.0205 (8)	0.0255 (10)	0.0215 (9)	0.0041 (8)	−0.0007 (7)	−0.0002 (8)
C1	0.0185 (9)	0.0115 (10)	0.0191 (9)	−0.0028 (8)	−0.0032 (8)	0.0023 (8)
C2	0.0148 (8)	0.0128 (9)	0.0257 (10)	−0.0009 (7)	0.0016 (8)	0.0030 (8)
C3	0.0214 (10)	0.0171 (10)	0.0198 (9)	−0.0040 (8)	0.0033 (8)	−0.0019 (9)
C4	0.0209 (9)	0.0180 (11)	0.0208 (10)	−0.0039 (8)	−0.0048 (8)	0.0027 (8)
C5	0.0158 (8)	0.0159 (9)	0.0247 (10)	−0.0013 (7)	−0.0035 (8)	0.0022 (10)
C6	0.0192 (9)	0.0154 (10)	0.0200 (10)	−0.0006 (8)	0.0018 (8)	0.0010 (8)
C7	0.0127 (8)	0.0189 (11)	0.0191 (9)	0.0027 (8)	0.0016 (7)	0.0009 (8)
C8A	0.017 (6)	0.0232 (14)	0.0196 (17)	0.0038 (12)	0.002 (3)	0.0022 (11)
O9A	0.039 (2)	0.032 (3)	0.025 (2)	0.0024 (17)	−0.0090 (14)	−0.0029 (16)
C9A	0.0214 (10)	0.018 (2)	0.018 (3)	0.0009 (13)	−0.009 (2)	−0.004 (2)
C10A	0.0299 (14)	0.036 (3)	0.023 (3)	0.002 (2)	−0.0067 (18)	−0.005 (2)
C11A	0.034 (2)	0.042 (3)	0.0184 (14)	0.006 (2)	−0.0097 (13)	−0.001 (2)
C8B	0.017 (6)	0.0232 (14)	0.0196 (17)	0.0038 (12)	0.002 (3)	0.0022 (11)
O9B	0.039 (2)	0.032 (3)	0.025 (2)	0.0024 (17)	−0.0090 (14)	−0.0029 (16)
C9B	0.0214 (10)	0.018 (2)	0.018 (3)	0.0009 (13)	−0.009 (2)	−0.004 (2)
C10B	0.0299 (14)	0.036 (3)	0.023 (3)	0.002 (2)	−0.0067 (18)	−0.005 (2)
C11B	0.034 (2)	0.042 (3)	0.0184 (14)	0.006 (2)	−0.0097 (13)	−0.001 (2)

### Geometric parameters (Å, º)

**Table:**

O1—N1	1.216 (2)	C5—H5	0.9500
O2—N1	1.223 (2)	C7—C8A	1.453 (7)
O3—N2	1.223 (2)	C7—C8B	1.471 (17)
O4—N2	1.215 (2)	C8A—C9A	1.352 (7)
O5—N3	1.223 (2)	C8A—O9A	1.372 (6)
O6—N3	1.218 (2)	O9A—C11A	1.400 (5)
O7—C7	1.371 (2)	C9A—C10A	1.439 (5)
O7—C1	1.377 (2)	C9A—H9	0.9500
O8—C7	1.198 (2)	C10A—C11A	1.348 (5)
N1—C2	1.479 (2)	C10A—H10	0.9500
N2—C4	1.473 (2)	C11A—H11	0.9500
N3—C6	1.468 (2)	C8B—C9B	1.345 (18)
C1—C6	1.391 (3)	C8B—O9B	1.376 (10)
C1—C2	1.395 (3)	O9B—C11B	1.388 (9)
C2—C3	1.383 (3)	C9B—C10B	1.365 (12)
C3—C4	1.381 (3)	C9B—H9B	0.9500
C3—H3	0.9500	C10B—C11B	1.344 (13)
C4—C5	1.383 (3)	C10B—H10B	0.9500
C5—C6	1.381 (3)	C11B—H11B	0.9500
			
C7—O7—C1	117.29 (15)	O7—C7—C8A	110.1 (4)
O1—N1—O2	123.95 (16)	O8—C7—C8B	128.7 (12)
O1—N1—C2	119.09 (16)	O7—C7—C8B	108.1 (10)
O2—N1—C2	116.96 (16)	C9A—C8A—O9A	113.0 (5)
O4—N2—O3	124.75 (18)	C9A—C8A—C7	131.2 (6)
O4—N2—C4	117.84 (17)	O9A—C8A—C7	115.8 (6)
O3—N2—C4	117.40 (18)	C8A—O9A—C11A	103.9 (5)
O6—N3—O5	124.81 (17)	C8A—C9A—C10A	105.1 (4)
O6—N3—C6	118.03 (17)	C8A—C9A—H9	127.5
O5—N3—C6	117.13 (17)	C10A—C9A—H9	127.5
O7—C1—C6	118.28 (17)	C11A—C10A—C9A	106.9 (4)
O7—C1—C2	123.73 (17)	C11A—C10A—H10	126.6
C6—C1—C2	117.64 (17)	C9A—C10A—H10	126.6
C3—C2—C1	121.45 (17)	C10A—C11A—O9A	111.1 (4)
C3—C2—N1	116.71 (17)	C10A—C11A—H11	124.5
C1—C2—N1	121.77 (17)	O9A—C11A—H11	124.5
C4—C3—C2	118.07 (18)	C9B—C8B—O9B	114.8 (15)
C4—C3—H3	121.0	C9B—C8B—C7	117.3 (16)
C2—C3—H3	121.0	O9B—C8B—C7	127.7 (18)
C3—C4—C5	123.00 (18)	C8B—O9B—C11B	103.2 (13)
C3—C4—N2	119.04 (18)	C8B—C9B—C10B	101.5 (13)
C5—C4—N2	117.95 (17)	C8B—C9B—H9B	129.3
C6—C5—C4	117.01 (16)	C10B—C9B—H9B	129.3
C6—C5—H5	121.5	C11B—C10B—C9B	113.3 (13)
C4—C5—H5	121.5	C11B—C10B—H10B	123.4
C5—C6—C1	122.59 (18)	C9B—C10B—H10B	123.4
C5—C6—N3	117.46 (16)	C10B—C11B—O9B	107.1 (13)
C1—C6—N3	119.95 (17)	C10B—C11B—H11B	126.5
O8—C7—O7	123.08 (17)	O9B—C11B—H11B	126.5
O8—C7—C8A	126.8 (4)		
			
C7—O7—C1—C6	−105.5 (2)	C1—O7—C7—O8	−1.9 (3)
C7—O7—C1—C2	81.4 (2)	C1—O7—C7—C8A	177 (2)
O7—C1—C2—C3	170.67 (19)	C1—O7—C7—C8B	−180 (6)
C6—C1—C2—C3	−2.5 (3)	O8—C7—C8A—C9A	−176 (3)
O7—C1—C2—N1	−6.0 (3)	O7—C7—C8A—C9A	5 (6)
C6—C1—C2—N1	−179.10 (17)	C8B—C7—C8A—C9A	−55 (71)
O1—N1—C2—C3	−177.87 (19)	O8—C7—C8A—O9A	3 (5)
O2—N1—C2—C3	2.0 (3)	O7—C7—C8A—O9A	−175 (3)
O1—N1—C2—C1	−1.1 (3)	C8B—C7—C8A—O9A	125 (80)
O2—N1—C2—C1	178.8 (2)	C9A—C8A—O9A—C11A	−2 (4)
C1—C2—C3—C4	−1.2 (3)	C7—C8A—O9A—C11A	179 (3)
N1—C2—C3—C4	175.64 (18)	O9A—C8A—C9A—C10A	2 (4)
C2—C3—C4—C5	1.8 (3)	C7—C8A—C9A—C10A	−178 (4)
C2—C3—C4—N2	−176.63 (19)	C8A—C9A—C10A—C11A	−2 (3)
O4—N2—C4—C3	164.0 (2)	C9A—C10A—C11A—O9A	0.8 (6)
O3—N2—C4—C3	−15.7 (3)	C8A—O9A—C11A—C10A	0 (2)
O4—N2—C4—C5	−14.5 (3)	O8—C7—C8B—C9B	−174 (6)
O3—N2—C4—C5	165.8 (2)	O7—C7—C8B—C9B	4 (13)
C3—C4—C5—C6	1.3 (3)	C8A—C7—C8B—C9B	125 (87)
N2—C4—C5—C6	179.71 (18)	O8—C7—C8B—O9B	12 (18)
C4—C5—C6—C1	−5.2 (3)	O7—C7—C8B—O9B	−170 (11)
C4—C5—C6—N3	173.92 (19)	C8A—C7—C8B—O9B	−49 (64)
O7—C1—C6—C5	−167.74 (19)	C9B—C8B—O9B—C11B	2 (12)
C2—C1—C6—C5	5.8 (3)	C7—C8B—O9B—C11B	176 (11)
O7—C1—C6—N3	13.2 (3)	O9B—C8B—C9B—C10B	−3 (12)
C2—C1—C6—N3	−173.29 (18)	C7—C8B—C9B—C10B	−178 (9)
O6—N3—C6—C5	−142.3 (2)	C8B—C9B—C10B—C11B	4 (7)
O5—N3—C6—C5	35.7 (3)	C9B—C10B—C11B—O9B	−3 (2)
O6—N3—C6—C1	36.8 (3)	C8B—O9B—C11B—C10B	1 (7)
O5—N3—C6—C1	−145.2 (2)		

### Hydrogen-bond geometry (Å, º)

**Table:**

*D*—H···*A*	*D*—H	H···*A*	*D*···*A*	*D*—H···*A*
C5—H5···O8^i^	0.95	2.32	3.270 (2)	180

Symmetry code: (i) −*x*+1, *y*−1/2, −*z*+1/2.
